# Access to Oncology Medicines in Canada: Consensus Forum for Recommendations for Improvement †

**DOI:** 10.3390/curroncol31040136

**Published:** 2024-03-29

**Authors:** Sandeep R. Sehdev, Nigel S. B. Rawson, Olexiy I. Aseyev, Catriona J. Buick, Marcus O. Butler, Scott Edwards, Sharlene Gill, Joanna M. Gotfrit, Cyrus C. Hsia, Rosalyn A. Juergens, Mita Manna, Joy S. McCarthy, Som D. Mukherjee, Stephanie L. Snow, Silvana Spadafora, David J. Stewart, Jason R. Wentzell, Ralph P. W. Wong, Pawel G. Zalewski

**Affiliations:** 1 Department of Medicine, Division of Medical Oncology, The Ottawa Hospital Cancer Centre, Ottawa, ON K1H 8L6, Canada; 2 Macdonald-Laurier Institute, Ottawa, ON K1N 7Z2, Canada; eastlakerg@gmail.com; 3 Thunder Bay Regional Health Sciences Centre, Thunder Bay, ON P7B 6V4, Canada; 4 Faculty of Health, York University, Toronto, ON M3J 1P3, Canada; 5 Odette Cancer Centre, Sunnybrook Health Sciences Centre, Toronto, ON M4N 3M5, Canada; 6 Canadian Association of Nurses in Oncology/Association Canadienne des Infirmières en Oncologie, Vancouver, BC V6A 1B6, Canada; 7 Princess Margaret Cancer Centre, University Health Network, Toronto, ON M5G 2C4, Canada; 8 Dr H Bliss Murphy Cancer Centre, Eastern Health, St. John’s, NL A1B 3V6, Canada; 9 School of Pharmacy, Memorial University of Newfoundland, St. John’s, NL A1B 3V6, Canada; 10 Department of Medicine, Division of Medical Oncology, University of British Columbia and BC Cancer, Vancouver, BC V5Z 1M9, Canada; 11 Department of Medicine, Division of Hematology, London Health Sciences Centre, London, ON N6A 5W9, Canada; 12 Juravinski Cancer Centre, McMaster University, Hamilton, ON L8V 5C2, Canada; 13 Saskatoon Cancer Centre, Saskatoon, SK S7N 4H4, Canada; 14 Cancer Care Program, Newfoundland and Labrador Health Services, St. John’s, NL A1B 3V6, Canada; 15 Dalhousie University, Halifax, NS B3H 2Y9, Canada; 16 Algoma District Cancer Program, Sault Area Hospital, Sault Ste Marie, ON P6B 0A8, Canada; 17 Northern Ontario School of Medicine University, Sudbury, ON P3E 2C6, Canada; 18 Department of Pharmacy, the Ottawa Hospital Cancer Centre, Ottawa, ON K1H 8L6, Canada; 19 CancerCare Manitoba, University of Manitoba, Winnipeg, MB R3E 0V9, Canada; 20 Lakeridge Health, Durham Regional Cancer Centre, Oshawa, ON L1G 8A2, Canada

**Keywords:** oncology drugs, health technology assessment, drug prices, drug access, Canada

## Abstract

Patient access to new oncology drugs in Canada is only possible after navigating multiple sequential systemic checkpoints for national regulatory approval, health technology assessment (HTA) and collective government price negotiation. These steps delay access and prevent health care providers from being able to prescribe optimal therapy. Eighteen Canadian oncology clinicians from the medicine, nursing and pharmacy professions met to develop consensus recommendations for defining reasonable government performance standards around process and timeliness to improve Canadian cancer patients’ access to best care. A modified Delphi methodology was used to identify consensus on 30 questions involving five themes: accountability, disparities, endpoints, timeliness, and cost-effectiveness. It was agreed that greater transparency is required across regulatory and HTA processes. Health professionals in oncology are frustrated for their patients because they are unable to deliver the modern guideline-supported therapies they want to provide due to delays in approval or funding. Canadian health care providers request improvements in timely access to life-saving therapeutics in line with other comparator countries. Clinicians expect urgent improvements in Canadian health systems to give our patients their best chance of survival.

## 1. Introduction

Cancer continues to be the leading cause of mortality in Canada. Two in five Canadians are expected to be diagnosed with cancer in their lifetime and approximately one in four will die from the disease [1]. New treatments continue to improve survival for many cancers, but they are only beneficial if patients have timely access. For many years, most (but not all) new cancer treatments were incorporated into Canadian health care systems. For example, between 2006 and 2014, just under 90% of new oncology drugs submitted for regulatory review in the United States and/or the European Union were submitted to Health Canada as well. However, the percentage of oncology medicines approved in the United States or the European Union coming to Canada steadily decreased from an average of 86% between 2006 and 2014 to only 50% in 2020 [2] when Patented Medicine Prices Review Board (PMPRB) reforms were proposed that would drastically reduce Canadian drug pricing [3,4,5,6]. These reforms were well intended but ill-considered and soundly rejected after national uproar, using unrealistic cost thresholds and not considering the special issues facing drugs for cancer or rare disorders. They served to discourage drug launches in Canada and clinical trial availability. The PMPRB has had to reconsider and is now planning a more collaborative and realistic approach.

Even if new oncology drugs are submitted and approved by Health Canada, their developers must overcome multiple barriers. Health technology assessments (HTA) are performed by the Institut national d’excellence en santé et en services sociaux (INESSS) for the province of Quebec [7] and the Canadian Institute for Drugs and Technologies in Health (CADTH) for the rest of Canada [8]). HTA reviews demand a low cost-effectiveness threshold of CAN$50,000 per quality-adjusted life year, an arbitrary value not adjusted for inflation since the 1980s [9]. Subsequent collective government drug plan price negotiations occur at the pan-Canadian Pharmaceutical Alliance (pCPA) [10]. All were established by Canadian federal, provincial and territorial governments and are responsible to them. Furthermore, drugs that successfully pass these barriers are not necessarily approved by these governments for reimbursement coverage in their drug plans [11] and, in some provinces, patients must pay significant copayments for oncology drugs [12].

Many new oncology therapies now in use or on the pharmaceutical horizon are targeted treatments for cancers caused by genetic mutations that can advance treatment beyond traditional options of surgery, radiation and chemotherapy [13]. These new drugs are often expensive and the obstacles created by Canadian governments frequently delay or deny patient access to them [11,14,15,16], prevent health care providers from prescribing optimal therapy, cause concern among patients and their families, and lead to lives being lost [17]. If Canadians with cancers are to fully benefit from new advanced treatments, current processes need to change [13]. While we cannot expect to use or fund every new treatment, Canadians should expect access comparable to other similar Organization for Economic Cooperation and Development (OECD) nations and decisions to be reached within reasonable timelines. However, in 2010–2014, Canada was fourth from the bottom out of 21 OECD nations in delivering timely cancer drug access [18].

The steps that new drugs must go through before health care providers can prescribe them and patients access them through public drug plans may create a robust and thorough review process. However, it is at the expense of delaying publicly funded access through government drug plans by one and a half to two years [19].

## 2. Materials and Methods

A meeting to determine the level of consensus around recommendations for actions to improve access to cancer medicines was held between 18 Canadian experts in oncology from the medicine, nursing and pharmacy professions from six provinces treating a broad variety of cancer types. Selected participants were actively practising in Canada, had some baseline knowledge of Canadian drug approval and reimbursement processes, were available to participate in pre-work and an in-person meeting, and constituted a broad representation of experts across common cancer types and geographic regions. There were no criteria to exclude interested parties from involvement.

A two-step modified Delphi methodology was used [20,21]. The first step was an online questionnaire developed by the lead author consisting of 38 questions. The questions included background information about participants’ years in practice, their understanding of Canada’s public drug reimbursement processes, whether any of their patients experience difficulty accessing cancer treatments, what proportion of their patients experience these challenges, their awareness of cancer therapies approved by Health Canada not being reimbursed by their province, and whether they had provided formal input to Health Canada, CADTH/INESSS, the pCPA, their province or a local therapeutics committee.

The remaining 30 questions were divided into five themes as listed in Table 1:

1. Accountability within the system between regulatory approval of a new oncology medicine through to public funding of the medicine (10 questions). Questions 1.2 to 1.8 and 1.10 required responses on a traditional five-point Likert scale (strongly agree, agree, neither agree nor disagree, disagree, or strongly disagree). For the other two questions (1.1 and 1.9), participants were asked to suggest appropriate performance standards for actions by governments and the pCPA.
2. Disparities within and across the system (5 questions). All questions but one (2.1, 2.2, 2.4 and 2.5) required responses on a Likert scale. Question 2.3 asked participants to suggest an appropriate performance target for the pCPA.
3. Endpoints that should be accepted within the system (7 questions). Five questions (3.1 to 3.5) required responses on a modified five-point Likert scale (strongly agree, agree, agree in some cases—consider clinician input, disagree, or strongly disagree), while the other two (3.6 and 3.7) required responses on the traditional Likert scale.
4. Timely access to new oncology medicines (3 questions). Two questions (4.1 and 4.2) required responses on a Likert scale. The third (4.3) asked what criteria should be used for inclusion in timely access to new medicines (novel therapies, treatments for rare malignancies, therapies where unmet need exists, or other criteria).
5. Cost-effectiveness within the system (5 questions). All five questions required answers on a Likert scale.

**Table 1:** The 30 Questions.

Theme 1: Accountability in the System
1.1 Federal/provincial/territorial jurisdictional silos are impediments to efficient and rapid drug access. Currently the usual time delay is about two (2) years. Governmental agreement for acceptable performance standards is required to achieve maximal delays of: (a) 3 months; (b) 6 months; (c) 9 months; (d) 12 months; (e) 18 months
1.2 CADTH should request for clinician input from all medical oncologists with expertise in a given malignancy. Provincial disease site group leads should solicit input from the broader treating clinician community before submitting recommendations. (a) Strongly agree; (b) Agree; (c) Neither agree nor disagree; (d) Disagree; (e) Strongly disagree
1.3 For cancer, CADTH should allow individual clinician input because cancer centres may have few experts in a malignancy and it can be daunting to coordinate inputs across Canada in the tight timeframes allowed. (a) Strongly agree; (b) Agree; (c) Neither agree nor disagree; (d) Disagree; (e) Strongly disagree
1.4 Upon receipt of clinician input, CADTH should attempt to arrange in-person discussions with at least five clinicians to review identified areas of concern. (a) Strongly agree; (b) Agree; (c) Neither agree nor disagree; (d) Disagree; (e) Strongly disagree
1.5 Final CADTH recommendations should highlight clinician and patient group inputs and specifically address them when disagreement exists about a new therapy. (a) Strongly agree; (b) Agree; (c) Neither agree nor disagree; (d) Disagree; (e) Strongly disagree
1.6 Health Canada, CADTH, INESSS and the pCPA should all formally recognize cancer as an exceptional disease and codify plans for expedited and innovative approval processes. (a) Strongly agree; (b) Agree; (c) Neither agree nor disagree; (d) Disagree; (e) Strongly disagree
1.7 Even common cancers are increasingly recognized as constituting a group of individually uncommon types driven by unique genomic drivers. While many cancers qualify as rare disorders, subtypes of common cancers do not. Individually distinct subtypes of cancers should be recognized as rare disorders allowing for the application of CADTH’s rare disease framework criteria for assessment, which should be consistently and rigorously applied. (a) Strongly agree; (b) Agree; (c) Neither agree nor disagree; (d) Disagree; (e) Strongly disagree
1.8 CADTH and the pCPA have become quasi-judicial bodies subject to legal review. Regular quality audits should be performed to review the key tenets of accessibility, affordability and appropriateness, and results published. (a) Strongly agree; (b) Agree; (c) Neither agree nor disagree; (d) Disagree; (e) Strongly disagree
1.9 After a positive clinical recommendation from CADTH, drugs proceed to the pCPA for pricing negotiations. Despite pCPA goals of initiating negotiations with 3 months, most oncology drugs routinely sit at their desk for six (6) months or longer before their processes even begin. The pCPA should formally recognize cancer as an exceptional disease requiring initiation of negotiations within: (a) 1 month; (b) 2 months; (c) 3 months; (d) 6 months; (e) No mandated performance standard
1.10 Cancer research is advancing very rapidly. Drugs may be approved or rejected based on early impressive clinical trial data. Further data may ensue very shortly thereafter requiring urgent updating of a previous decision. However, Health Canada and CADTH require entirely new submissions. For cancer drugs, Health Canada and CADTH processes should be modernized, allowing for “rolling reviews” (i.e., rapid updates on previous assessments based on updated data). (a) Strongly agree; (b) Agree; (c) Neither agree nor disagree; (d) Disagree; (e) Strongly disagree
**Theme 2: Disparities in Access in the System**
2.1 CADTH and the pCPA strive to standardize health technology assessment and pricing across Canada. However, approval decisions frequently differ between INESSS and CADTH. The processes should be aligned and CADTH’s process should include assessment of societal values (as INESSS does). (a) Strongly agree; (b) Agree; (c) Neither agree nor disagree; (d) Disagree; (e) Strongly disagree
2.2 CADTH and INESSS should integrate their processes, initially through cooperation and consultation defaulting to recommending a new therapy if either body does. (a) Strongly agree; (b) Agree; (c) Neither agree nor disagree; (d) Disagree; (e) Strongly disagree
2.3 Post-pCPA, provinces/territories should decide on funding within: (a) 1 month; (b) 2 months; (c) 3 months; (d) 6 months; (e) No mandated performance standard
2.4 Canada has a multi-tiered health care system, with recognized disparities in access to many medical technologies and treatments based on socio-economic factors and income. Inequities in access to cancer treatments is, however, unacceptable. All treatments should be provided by cancer clinics. (a) Strongly agree; (b) Agree; (c) Neither agree nor disagree; (d) Disagree; (e) Strongly disagree
2.5 Many patients require proven and approved cancer drugs that are not yet funded publicly. They require complex assistance from drug navigators (reimbursement coordinators), which have been required for over a decade and are usually paid for by individual hospitals. Many hospitals cannot afford any such navigators or do not have enough. Provincial and territorial governments and cancer programs should specifically fund drug navigator positions. (a) Strongly agree; (b) Agree; (c) Neither agree nor disagree; (d) Disagree; (e) Strongly disagree
**Theme 3: Endpoints Acceptability**
Surrogate endpoints should be accepted, when supported by guidelines or clinical consensus, including:
3.1 Progression free survival. (a) Strongly agree; (b) Agree; (c) Agree in some cases—consider clinician input; (d) Disagree; (e) Strongly disagree
3.2 Disease free or event free survival in adjuvant studies. (a) Strongly agree; (b) Agree; (c) Agree in some cases—consider clinician input; (d) Disagree; (e) Strongly disagree
3.3 Quality of life and symptom benefit. (a) Strongly agree; (b) Agree; (c) Agree in some cases—consider clinician input; (d) Disagree; (e) Strongly disagree
3.4 Response rates including complete response. (a) Strongly agree; (b) Agree; (c) Agree in some cases—consider clinician input; (d) Disagree; (e) Strongly disagree
3.5 Phase 2 trials, especially randomized phase 2 trials, sometimes demonstrate dramatic efficacy and should be accepted as evidence when the magnitude of benefit warrants or where confirmatory phase 3 trials may not be possible. (a) Strongly agree; (b) Agree; (c) Agree in some cases—consider clinician input; (d) Disagree; (e) Strongly disagree
3.6 Molecular or biologic endpoints, such as minimal residual disease assessments and circulating tumour DNA, have become accepted as markers of residual disease, response to therapy, development of resistance to therapy, and markers of genomic evolution to guide therapy changes. Canada’s regulatory and health technology assessment processes should be updated and adaptive to rapidly emerging science in this area. (a) Strongly agree; (b) Agree; (c) Neither agree nor disagree; (d) Disagree; (e) Strongly disagree
3.7 High-quality real-world evidence is now possible to support promising early data while randomized trials are underway or to provide data where phase 3 randomized clinical trials for overall survival are not feasible. Governments should support development of real-world evidence where needed or desirable. (a) Strongly agree; (b) Agree; (c) Neither agree nor disagree; (d) Disagree; (e) Strongly disagree
**Theme 4: Timely Access to Treatment**
4.1 In Canada, consistent early managed access programs, such as CADTH’s Time Limited Recommendation, are needed immediately to allow for equitable publicly funded access to breakthrough cancer therapies. (a) Strongly agree; (b) Agree; (c) Neither agree nor disagree; (d) Disagree; (e) Strongly disagree
4.2 For drugs denied funding after health technology review, Canadian clinicians would accept withdrawal of funding for subsequent patients. (a) Strongly agree; (b) Agree; (c) Neither agree nor disagree; (d) Disagree; (e) Strongly disagree
4.3 Criteria for eligibility should include (select all that apply). (a) Novel therapies; (b) Rare malignancies; (c) Unmet need; (d) Other (please describe)
**Theme 5: Cost-effectiveness in the System**
5.1 Future PMPRB processes need to recognize cancer and rare diseases as special circumstance with separate pathways, which should not require additional time beyond existing steps. (a) Strongly agree; (b) Agree; (c) Neither agree nor disagree; (d) Disagree; (e) Strongly disagree
5.2 PMPRB restrictions will limit Canadian patients’ access to clinical trials and compassionate access programs. (a) Strongly agree; (b) Agree; (c) Neither agree nor disagree; (d) Disagree; (e) Strongly disagree
5.3 The current cutoff health technology assessment threshold of $50,000/quality-adjusted life year gained is arbitrary, based on other medical conditions, and has never adjusted for inflation since it was proposed in the 1980s nor for the much higher costs of drug development today. The threshold is unreasonable and unacceptable to clinicians and patients. The previous threshold of $100,000/quality-adjusted life year should be reinstated and higher amounts considered where appropriate. (a) Strongly agree; (b) Agree; (c) Neither agree nor disagree; (d) Disagree; (e) Strongly disagree
5.4 Shared risk models. Although complex, such models work effectively in Europe. Governments can demand repayment for drugs that underperform in Canada. Pay for performance/risk sharing agreements should be sought by the pCPA for new drugs. (a) Strongly agree; (b) Agree; (c) Neither agree nor disagree; (d) Disagree; (e) Strongly disagree
5.5 Rebates/reimbursements. Health care systems are strained across Canada, especially in oncology. Negotiated rebates or repayments (risk sharing) currently go back into provincial coffers but not back into health care budgets. They should be reinvested into provincial cancer drug programs. (a) Strongly agree; (b) Agree; (c) Neither agree nor disagree; (d) Disagree; (e) Strongly disagree

CADTH: Canadian Agency for Drugs and Technologies in Health; INESSS: Institut national d’excellence en santé et en services sociaux; pCPA: pan-Canadian Pharmaceutical Alliance; PMPRB: Patented Medicine Prices Review Board.

Subsequently, the same 18 experts met in person on 16 September 2023, in a facilitated session. The threshold for consensus agreement was set at 80% or higher. Stopping rules were pre-determined such that a recommendation was discarded if less than 80% consensus was found (i.e., fewer than 80% of the votes agreeing or strongly agreeing).

It was emphasized to participants that the objective was to arrive at reasonable consensus recommendations to address the delays in Canadian drug access processes that presently threaten patient survival and quality of life. The purpose of the process was *not* to get particular drugs or “every drug” funded in Canada, but rather to mandate reasonable processes that serve our patients’ needs and recognize the true urgency and gravity of their situation.

The session was conducted by a live facilitator (Erik Lockhart, Queen’s University Decision Centre) coordinating active verbal interactions and using an electronic meeting system with a state-of-the-art group decision support system to enable participants to rapidly accelerate idea generation and consensus building using a network of laptop computers. When participants were asked a question, they entered their rating and could add anonymized ideas electronically.

An objective of the participants in the session was to gain external support for the results of the Delphi process from national organizations representing key professionals and patients. A draft of this manuscript was sent to the Canadian Association of Medical Oncologists, the Canadian Association of Nurses in Oncology/Association canadienne des infirmières en oncologie, the Canadian Association of General Practitioners in Oncology, the Society of Gynecologic Oncology of Canada, and the CanCertainty Coalition (the united voice of more than 30 Canadian patient groups, cancer health charities and caregiver organizations working with oncologists and cancer care professionals to improve the affordability and accessibility of cancer treatment).

## 3. Results

### 3.1. Participant Information

The 18 participants comprised 15 medical oncologists, 2 lead oncology pharmacists and 1 oncology nurse representing a national organization, all of whom treat patients and hold academic or community leadership posts. They practise in Ontario (12; 66.7%), the Western provinces of British Columbia, Saskatchewan and Manitoba (3; 16.7%) and the Atlantic provinces of Nova Scotia and Newfoundland and Labrador (3; 16.7%). Most (11; 61.1%) had been in practice for 20 years or more; four (22.2%) had practised for six to 19 years and the other three (16.7%) for five years or less.

Two-thirds considered themselves to have a good or excellent understanding of Canada’s public drug reimbursement processes, while the other third have an average understanding. More than 60% of the experts had provided formal input to their province and a local therapeutics committee in the past year and over 80% had provided input to CADTH and/or INESSS. However, under 10% had provided formal input to Health Canada or the pCPA.

Over 93% of the experts reported patients in their practice experiencing difficulty with cancer treatment access—20% said many patients per week, 40% many per month, and 33% a few patients per month. Most of the experts (60%) reported that up to a quarter of their patients experience drug reimbursement challenges and over a quarter reported 26–50% of their patients experiencing such challenges. More than 93% are aware of a cancer therapy approved by Health Canada but not reimbursed in their province.

### 3.2. Theme 1—Accountability in the System

The 80% or higher consensus agreement level (strongly agree or agree) was achieved for seven of the eight Likert scale questions (Table 2A). The other two questions required participants to consider time frames for funding decisions and initiation of negotiations after reimbursement recommendation for an oncology medicine; 88.9% of the participants agreed that a maximal performance standard for time from evidence to a funding decision should be six months or less (currently 2–4 years [11]) and 94.4% wanted the pCPA to formally recognize cancer as an exceptional disease requiring initiation of negotiations within one month of a positive HTA reimbursement recommendation (Table 2B). One participant commented that current delays are unacceptable and “there should be novel methods for rapid access” after regulatory approval. Another stated that HTAs should be part of Health Canada’s assessment.

The participants had much to say about clinician input into CADTH reviews, with one stating that they should “as a discipline confirm who is chosen to provide this input” and another commenting that broader clinician input would help avoid bias. Question 1.5, which asked whether they supported final CADTH reimbursement recommendations should highlight clinician and patient group inputs and specifically address them when disagreement exists about a new therapy, stimulated several comments, including:

- “Clinician input is mentioned [in HTA reports], but I feel that the rigor of applying an evaluation framework is hit or miss”.
- “Documenting clinician and patient input in the final recommendation would demonstrate transparency and inclusivity of opinions outside the CADTH committee”.
- “Transparency is crucial to understand the ultimate decision and, in the setting of disagreement, specific reasons why the expert review committee’s conclusions were considered more relevant than other(s) should be documented”.

Question 1.6 asking whether Health Canada, CADTH/INESSS and the pCPA should all formally recognize cancer as an exceptional disease and develop plans for expedited and innovative approval processes elicited a particularly powerful comment from one participant: “We and others have published on the thousands of life-years that may be lost due to delays in the availability of effective new therapies. In addition, patients may rapidly die while we do paperwork to try to access therapies that are approved but require individual approval for patients. For metastatic non-small cell lung cancer, 4% of remaining patients die during each week that therapy initiation is delayed”. Multiple participants mentioned the vital need for transparency in the system when asked whether regular published quality audits should be held to review CADTH’s performance around the key tenets of accessibility, affordability and appropriateness.

Responses to question 1.10 about whether Health Canada and CADTH should allow “rolling reviews” for cancer drugs indicated unanimous agreement. Cancer research is advancing very rapidly. Drugs may be approved or rejected based on early impressive clinical trial data, sometimes with restrictions to specific patient sub-populations. Further data may ensue very shortly thereafter (e.g., more mature, more data on subgroups, final survival outcomes, or supportive data from other trials) requiring urgent updating of a previous decision such as approving a previously denied drug or removing restrictions. However, unlike the rolling review approach taken in Europe and the United States approaches, Health Canada and CADTH require entirely new submissions—“starting from scratch” as one participant commented—which may delay Canadians’ access to therapy by more than an additional year. Another participant commented: “Since cancer is the leading cause of death, we must do better and be more efficient. Rolling reviews help to avoid delays”.

Question 1.3 concerning whether CADTH should allow individual clinician input because cancer centres may have few experts to coordinate group input produced wide variation in the voting. Even participants that supported individual input thought it made sense for rare cancers but not necessarily for more common tumours. On the other hand, a participant strongly disagreed with individual input because they had seen a situation where one clinician’s negative input had swayed a recommendation despite “many other voices with a different opinion”.

### 3.3. Theme 2—Disparities in Access in the System

Four questions discussed disparities in access in the system (question 2.2 was considered by the participants to be made redundant by question 2.1). Consensus agreement (strongly agree or agree) was achieved for all three Likert scale questions (Table 3A). The participants strongly supported all treatments being provided by cancer clinics (94.4%) and that provincial and territorial governments should fund drug reimbursement navigator positions (100.0%). They also agreed that CADTH and INESSS processes for cancer drugs should be integrated and that CADTH should consider a drug’s societal value as INESSS does (94.4%). They favoured Canada having only one HTA process for cancer drugs because this would “increase efficiency and reduce indefensible disparity”.

Question 2.3 required participants to consider how quickly provinces and territories should decide on funding for a cancer drug following a successful pCPA price negotiation. Currently provincial decisions, after the multi-provincial pCPA process, can take anywhere from one to six months or more. The participants were strongly supportive (83.3%) of provinces and territories deciding on funding within one month (Table 3B).

### 3.4. Theme 3—Endpoints Acceptability

Five questions in this theme asked participants to vote on a modified Likert scale of strongly agree, agree, agree in some cases—consider clinician input, disagree, or strongly disagree about the acceptability of surrogate endpoints. Over 94% of the participants supported broader acceptance of progression-free survival (and disease- or event-free survival in neoadjuvant/adjuvant studies) in many settings where broad international consensus exists, while acknowledging challenges related to unvalidated surrogate endpoints (Table 4A). Participants were more reserved about the acceptance of quality of life and symptom benefit, response rates including complete response, and evidence from phase 2 trials. However, all participants agreed that these responses and evidence were a reasonable endpoint in at least some situations.

The remaining two questions in this theme used the traditional five-point Likert scale. Over 83% of the participants agreed or strongly agreed that Canada’s regulatory and HTA processes should be updated and adaptive to rapidly emerging medical science (such as molecular endpoints) and over 94% agreed or strongly agreed that governments should accept *and support* the development of real-world evidence (Table 4B).

Comments to questions in this theme were varied and highlighted the evolving complexity and technical nature of the topic However, participants’ comments suggested that Health Canada and CADTH reviews need, at least in some circumstances, to take account of surrogate endpoints to enhance patients’ access to innovative oncology treatments.

### 3.5. Theme 4—Timely Access to Treatment

Participants strongly agreed (83.3%) on the need for consistent early managed access programs in Canada to allow equitable publicly funded rapid access to breakthrough cancer therapies (Table 5). Participants commented that “there are existing examples/infrastructures in other countries that expedite access for patients”. They also suggested that Canada “needs to align with the rest of the world” because “quicker, smarter approaches are needed. We cannot be an outlier compared to the rest of the world. We need a system for immediate access and rapid review while formal HTA reviews are initiated to formally confirm the usefulness of the agent”. Over 94% of participants either strongly agreed (50.0%) or agreed (44.4%) that Canadian clinicians would accept the withdrawal of funding if the HTA review subsequently recommended a drug not be reimbursed.

All the participants thought therapies for rare malignancies and treatments where unmet needs exist should be eligible for early managed access programs and 94.4% thought that novel therapies should also be included. Other suggestions for eligibility were new indications, or oral or subcutaneous drugs allowing access for patients who are not close to a cancer centre.

### 3.6. Theme 5—Cost-Effectiveness in the System

The participants strongly agreed (83.3%) that future PMPRB processes need to recognize cancer and rare diseases as special circumstances with separate pathways (Table 6). Every participant strongly agreed that, for cancer drugs, the $50,000 per quality-adjusted life year (QALY) threshold for cost-effectiveness is unreasonable and unacceptable to clinicians and patients and a higher threshold should be reinstated (one participant commented that the “$50k/QALY is outdated and out of touch with reality”). The threshold was originally proposed in the 1980s for end-stage renal disease kidney dialysis cost-effectiveness and became widely used for the cost-effectiveness of medications in the 1990s [9]; it has never been updated for inflation, the complexity of new medicines, or the increase in drug prices.

In addition, 94.4% strongly agreed that pCPA-negotiated rebates should be reinvested in provincial cancer drug programs and not into general government accounts Over 83% of the participants agreed or strongly agreed that planned PMPRB restrictions will limit Canadian patients’ access to clinical trials [3,4,5,6] (one participant commented that “this is already a reality”) and compassionate access programs. There was consensus that risk-sharing agreements should be sought by the pCPA for new drugs. One participant stated “I think that [PMPRB] restrictions will limit Health Canada submissions and will stifle access to drugs and clinical trials. We need to avoid delay in launch dates”.

### 3.7. External Validation

The results are supported by the Canadian Association of Medical Oncologists, the Canadian Association of Nurses in Oncology/Association canadienne des infirmières en oncologie, the Society of Gynecologic Oncology of Canada, the Canadian Association of General Practitioners in Oncology and the CanCertainty Coalition (a coalition of more than 30 Canadian patient groups, cancer health charities and caregiver organizations).

## 4. Limitations

The objective of the Delphi process was to measure consensus amongst a range of health care providers involved in actually treating cancer with systemic therapies on how access to these treatments, which HTA specialists and politicians have admitted is not working in Canada, should be significantly improved. While 30 clinicians were invited to participate, 18 accepted and while it is possible that they may not represent all cancer clinicians in Canada (those motivated to improve access may have been more likely to participate) they do represent a wide spectrum of specialties and jurisdictions across Canada.

The results are not meant to present a balanced perspective on what can be done or can be afforded, but rather to demonstrate the consensus amongst cancer clinicians, who want to provide optimal care to their patients, around what they feel needs to be done in terms of performance standards, accountability, and achievement of published targets that are consistently not being met. The strong consensus demonstrated, while perhaps not surprising, is important to document to spur needed and long overdue process improvements. The conclusion that almost no cancer experts find the current status quo to be acceptable should come across loudly and clearly.

We did not address issues around what we are willing to trade off for improved and timely access to new oncology therapies. We acknowledge that the costs of oncology medicines are rising globally and further recognize that pharmaceutical companies have an important role in considering fair pricing strategies. We do not expect all oncology drugs to be funded in Canada and we recognize the importance of price negotiations. We further recognize that health care funding is limited and that there are many other calls for improved treatment in other areas of medicine. However, it is the responsibility of governments in Canada to delivery adequate health care but not necessarily to define what is adequate without important inputs from clinicians and patients in the context of what should be achievable health delivery standards, compared with other comparator nations of similar means. It is not the role of clinicians to come up with all of the solutions, but our work (and those referenced) have defined many of the problems and metrics demonstrating the failures of timely and equitable access to treatments in Canada and the negative impact on patients’ lives.

## 5. Discussion

Oncology drugs are medications given to patients who have cancer to cure in some cases or to modify their disease or symptoms, which may prolong their lives. Not all oncology drugs launched in the United States and the European Union are launched in Canada [19]. Those that do come to Canada are launched later than in the United States and/or Europe by a year or so [22,23]. Furthermore, a drug can have regulatory approval in Canada, but this does not necessarily mean it can be accessed by patients via their provincial or territorial drug plan for many months or even years, if at all [11]. After federal regulatory approval, developers must have their drug reviewed and recommended for reimbursement by the country’s HTA agencies (CADTH/INESSS), owned and funded by the federal, provincial and territorial governments, then be invited to negotiate with the same governments’ price bargaining organization (pCPA) and successfully achieve an agreed price. Subsequently, they negotiate with individual government drug plans to be listed in their formularies. These are sequential time- and resource-consuming processes that can deter some drug developers from bringing drugs or new indications for existing drugs to Canada—meaning no access for Canadian patients at all [24]. Other developers may only launch their drugs in Canada after doing so in other countries, leading to significant delays in access for Canadian patients. When the drugs in question are oncology drugs, lives are lost [1,17].

In the last decade, novel oncology therapies have been developed to target cancers more accurately than before [25,26]. The prices of these drugs are frequently high, which has led to attempts by the federal government to reduce drug prices in Canada. Canadian courts have ruled that the more drastic restrictions are unconstitutional [27]. Nevertheless, for several years, CADTH has regularly included a recommendation for a price reduction to allow the drug to be cost-effective at the threshold of $50,000 per QALY. The recommended percentage price reduction can be substantial. For example, in 55 recommendations for oncology medications issued by CADTH between January 2020 and December 2021, over 61% had a recommended price reduction of 70% or more [24]. Given the cost of drug development and global considerations, such reductions are unlikely to be agreed upon by manufacturers in pCPA negotiations and, consequently, government drug plans are faced with significant increases in their drug budgets. Canada is a federation and not one unitary jurisdiction, which results in some plans deciding not to fund drugs.

As participants at the facilitated session noted, health care providers are placed in a difficult situation when a drug is approved in Canada but not funded in their province. They are obligated to inform patients about recommendations for optimal treatment for their cancer but, if the public system does not reimburse the drug, they need to prescribe alternative suboptimal funded treatment instead. Meeting participants noted that it is not the health care provider’s role to get a drug paid for, although they can spend hours per patient completing paperwork to try to obtain funding for the best therapy. While they strongly agreed that provincial and territorial governments should fund drug reimbursement navigator positions to assist patients to try to gain access to medicines, this role is only a band-aid and not a solution to access problems.

Results from the facilitated session demonstrated consensus agreement (agree or strongly agree being 80% or higher) among the participants on almost all the accountability, disparities, access to treatment and cost-effective questions. Governments should take responsibility for explaining to their citizens why it takes so long to perform HTAs, why it can take many months for the pCPA to decide to negotiate prices for drugs with a reimbursement recommendation, why negotiations can take nearly a year [28], and why cancer therapies that other Canadians, Americans and Europeans can access are not available to them. The participants reached consensus agreement on the need for much greater and specific transparency in regulatory and HTA reviews as a crucial part of being accountable to patients and health care providers. They want to see specialized and meaningful clinical input from a wide range of relevant oncologists respected in HTA reviews.

The participants also agreed that Canada’s regulatory and HTA processes need to be updated and adaptive to emerging science. This includes more rapid HTA review processes and the use of “rolling reviews”. CADTH has announced that it is introducing time-limited drug reimbursement recommendations “that will aim to help provide earlier access to promising new treatments that target the unmet needs of people in Canada living with severe, rare, or debilitating illnesses” [29]. A time-limited recommendation to publicly fund a therapy will apply to those rare drugs given conditional approval by Health Canada (these are predominantly oncology products) for a specific period of time and require developers to conduct ongoing clinical studies to address uncertainty in the evidence. CADTH will conduct a future reassessment of the additional evidence, which will lead to a final reimbursement recommendation. Whether this will accelerate patient access is debatable. Health Canada and CADTH are also introducing “rolling reviews”, but they will only be applicable to COVID-19 drugs [30,31]. The lessons learned from the urgency around COVID-19 need to be applied to the even greater national menace of cancer.

Endpoint acceptability led to considerable discussion among the participants. They were in agreement about the acceptability in many cases of progression- and disease-free survival [32,33]. However, participants thought that the value of quality of life, response rates and phase 2 trial data was context dependent. They did agree that the regulatory and HTA processes should be adaptive to emerging science and the needs of patients. This topic requires a wider discussion among health professionals and regulatory and HTA agencies concerning which endpoints and what types of data are acceptable, in which circumstances they are acceptable, and how to ensure consistency in their application.

In their support for the results of the Delphi process, the CanCertainty Coalition congratulated the meeting participants on identifying the barriers that exist in accessing cancer treatment and achieving consensus around recommendations to improve access. The Coalition is completely aligned with the recommendations for improvement to Canada’s regulatory, HTA, price negotiation and drug plan decision-making processes.

The process challenges described are also under active review and revision internationally, with an increasing recognition of the urgency involved. Common themes in Europe, Australia and New Zealand include: streamlining/combining steps, developing rapid access programs before final HTA approvals, expanding the role of patient voices, improving transparency and measuring outcomes.

## 6. Conclusions

Cancer remains a leading cause of suffering and death in Canada. The facilitated meeting of oncology experts demonstrated that they are concerned that Canada’s regulatory, HTA, price negotiation and government drug plan decision-making processes take much too long, that they need greater accountability and transparency for patients, that they must include societal benefits of new medicines, that they should be adaptive to developments in emerging science, and that the current arbitrary $50,000 per QALY cost-effectiveness threshold used by CADTH must be increased. Without these and other changes, Canadian patients will continue to die because of a lack of timely access to innovative oncology therapies [17]. Health professionals in oncology are frustrated about their frequent inability to provide what they consider to be the best therapy for their patients due to delays in approval or funding. Clinicians want to see changes to the health system that would allow their patients timely access to medicines that would give them a fighting chance against cancer.

## Figures and Tables

**Table 2:** Accountability in the System (Theme 1).

A: Likert-Scale Questions	Strongly Agree	Agree	Neither Agree nor Disagree	Disagree	Strongly Disagree
1.2: CADTH should request input from all oncologists with expertise in a malignancy; provincial disease site group leads should solicit input from the broader treating community before submitting recommendations	14 (77.8%)	3 (16.7%)	0 (0.0%)	1 (5.6%)	0 (0.0%)
1.3: CADTH should allow individual clinician input because cancer centres may have few experts in a malignancy and it can be daunting to coordinate inputs across Canada in the tight timeframes allowed	6 (33.3%)	6 (33.3%)	2 (11.1%)	2 (11.1%)	2 (11.1%)
1.4: Upon receipt of clinician input, CADTH should attempt to arrange in-person/virtual discussions with at least five clinicians to review identified areas of concern	10 (55.6%)	7 (38.9%)	0 (0.0%)	1 (5.6%)	0 (0.0%)
1.5: Final CADTH recommendations should highlight clinician and patient group inputs and specifically address them when disagreement exists about a new therapy *	15 (88.2%)	2 (11.8%)	0 (0.0%)	0 (0.0%)	0 (0.0%)
1.6: Health Canada, CADTH/INESSS and pCPA should all formally recognize cancer as an exceptional disease and codify plans for expedited and innovative approval processes	16 (88.9%)	2 (11.1%)	0 (0.0%)	0 (0.0%)	0 (0.0%)
1.7: Distinct subtypes of cancers should be recognized as rare disorders allowing for application of CADTH’s rare disease framework criteria for assessment, which should be applied consistently and rigorously	15 (83.3%)	2 (11.1%)	0 (0.0%)	0 (0.0%)	1 (5.6%)
1.8: Regular quality audits should be performed to review the key tenets of accessibility, affordability and appropriateness, and results published	13 (72.2%)	5 (27.8%)	0 (0.0%)	0 (0.0%)	0 (0.0%)
1.10: For cancer drugs, Health Canada and CADTH processes should be modernized, allowing for “rolling reviews”	18 (100.0%)	0 (0.0%)	0 (0.0%)	0 (0.0%)	0 (0.0%)
**B: Time-period Questions**	**3 months**	**6 months**	**9 months**	**12 months**	**18 months**
1.1: Governmental agreement for acceptable performance standards is required to achieve maximal delays for drug access of:	5 (27.8%)	11 (61.1%)	1 (5.6%)	1 (5.6%)	0 (0.0%)
	**1 month**	**2 months**	**3 months**	**6 months**	**No standard**
1.9: The pCPA should formally recognize cancer as an exceptional disease requiring initiation of negotiations within:	17 (94.4%)	0 (0.0%)	0 (0.0%)	1 (5.6%)	0 (0.0%)

* One participant abstained.

**Table 3:** Disparities in Access in the System (Theme 2).

A: Likert-Scale Questions	Strongly Agree	Agree	Neither Agree nor Disagree	Disagree	Strongly Disagree
2.1: CADTH/INESSS processes should be integrated and include an assessment of societal values (as INESSS does)	13 (72.2%)	4 (22.2%)	1 (5.6%)	0 (0.0%)	0 (0.0%)
2.4: All treatments should be provided by cancer clinics	14 (77.8%)	3 (16.7%)	1 (5.6%)	0 (0.0%)	0 (0.0%)
2.5: Provincial and territorial governments and cancer programs should specifically fund drug navigator positions	18 (100.0%)	0 (0.0%)	0 (0.0%)	0 (0.0%)	0 (0.0%)
**B: Time-period Questions**	**1 month**	**2 months**	**3 months**	**6 months**	**No standard**
2.3: Post-pCPA, provinces/territories should decide on funding within:	15 (83.3%)	2 (11.1%)	1 (5.6%)	0 (0.0%)	0 (0.0%)

**Table 4:** Endpoints Acceptability (Theme 3).

A: Five-Point Scale Questions	Strongly Agree	Agree	Agree in Some Cases	Disagree	Strongly Disagree
Surrogate endpoints should be accepted, when supported by guidelines or clinical consensus, including					
3.1: Progression free survival *	12 (70.6%)	5 (29.4%)	0 (0.0%)	0 (0.0%)	0 (0.0%)
3.2: Disease free or event free survival in adjuvant studies	16 (88.9%)	1 (5.6%)	0 (0.0%)	1 (5.6%)	0 (0.0%)
3.3: Quality of life and symptom benefit *	7 (41.2%)	5 (29.4%)	4 (23.5%)	1 (5.9%)	0 (0.0%)
3.4: Response rates including complete response	4 (22.2%)	7 (38.9%)	7 (38.9%)	0 (0.0%)	0 (0.0%)
3.5: Phase 2 trials, especially randomized trials, sometimes demonstrate dramatic efficacy and should be accepted as evidence when the magnitude of benefit warrants or where confirmatory phase 3 trials may not be possible	9 (50.0%)	3 (16.7%)	6 (33.3%)	0 (0.0%)	0 (0.0%)
**B: Likert-scale Questions**	**Strongly agree**	**Agree**	**Neither agree nor disagree**	**Disagree**	**Strongly disagree**
3.6: Canada’s regulatory and health technology assessment processes should be updated and adaptive to rapidly emerging science in molecular or biologic endpoints	10 (55.6%)	5 (27.8%)	3 (16.7%)	0 (0.0%)	0 (0.0%)
3.7: Governments should support development of real-world evidence where needed or desirable	11 (61.1%)	6 (33.3%)	1 (5.6%)	0 (0.0%)	0 (0.0%)

* One participant abstained.

**Table 5:** Timely Access to Treatment (Theme 4).

Likert-Scale Questions	Strongly Agree	Agree	Neither Agree nor Disagree	Disagree	Strongly Disagree
4.1: In Canada, consistent early managed access programs, such as CADTH’s Time Limited Recommendation, are needed immediately to allow for equitable publicly funded access to breakthrough cancer therapies	15 (83.3%)	2 (11.1%)	1 (5.6%)	0 (0.0%)	0 (0.0%)
4.2: For drugs denied funding after health technology review, Canadian clinicians would accept withdrawal of funding for subsequent patients	9 (50.0%)	8 (44.4%)	1 (5.6%)	0 (0.0%)	0 (0.0%)

**Table 6:** Cost-Effectiveness in the System (Theme 5).

Likert-Scale Questions	Strongly Agree	Agree	Neither Agree nor Disagree	Disagree	Strongly Disagree
5.1: Future PMPRB processes need to recognize cancer and rare diseases as special circumstance with separate pathways, which should not require additional time beyond existing steps	15 (83.3%)	3 (16.7%)	0 (0.0%)	0 (0.0%)	0 (0.0%)
5.2: PMPRB restrictions will limit Canadian patients’ access to clinical trials and compassionate access programs	10 (55.6%)	5 (27.8%)	3 (16.7%)	0 (0.0%)	0 (0.0%)
5.3: The $50,000/QALY threshold is unreasonable and unacceptable to clinicians and patients. The previous threshold of $100,000/QALY should be reinstated and higher amounts considered where appropriate	18 (100.0%)	0 (0.0%)	0 (0.0%)	0 (0.0%)	0 (0.0%)
5.4: Pay for performance/risk sharing agreements should be sought by the pCPA for new drugs	11 (61.1%)	7 (38.9%)	0 (0.0%)	0 (0.0%)	0 (0.0%)
5.5: Negotiated rebates or repayments (risk sharing) currently go back into provincial coffers but not into health care budgets. They should be reinvested into provincial cancer drug programs	17 (94.4%)	1 (5.6%)	0 (0.0%)	0 (0.0%)	0 (0.0%)

## Data Availability

All data are shown in the tables.
